# Leveraging Clinical Decision Support System Tools for Childhood Overweight/Obesity Management

**DOI:** 10.21203/rs.3.rs-7564927/v1

**Published:** 2025-10-03

**Authors:** Joseph R. Wardell, Nora Shaska, Zara Ahmed, Brigid Gregg, Kanakadurga Singer, Jung Eun Lee, Emily Hirschfeld, Ashley Garrity, Susan Woolford, Karen E. Peterson, Jennifer Bragg-Gresham, Kelly Orringer, Lauren Oshman, Jonathan Gabison, Layla Mohammed, Esther Yoon, Jacob Bilhartz, Bonnie Burns, Joyce M. Lee

**Affiliations:** University of Michigan; University of Michigan; University of Michigan; University of Michigan; University of Michigan; University of Michigan; University of Michigan; University of Michigan; University of Michigan; University of Michigan School of Public Health; University of Michigan; University of Michigan; University of Michigan; University of Michigan; University of Michigan; University of Michigan; University of Michigan; University of Michigan; University of Michigan

**Keywords:** pediatric, OurPractice advisory, obesity, clinical decision support system

## Abstract

**Introduction:**

Consistent with evidence-based care for pediatric overweight (BMI between 85^th^ to 94^th^ percentile) and obesity (BMI ≥ 95^th^ percentile), a clinical decision support system (CDSS) OurPractice Advisory (OPA) was designed and implemented in 18 pediatric primary care clinics to support documenting elevated body mass index (BMI) on electronic health record (EHR) problem lists and ordering comorbidity screening labs.

**Methods:**

For those whom the OPA fired, we assessed the odds of problem list BMI documentation and comorbidity screening based on demographics and weight status and generated statistical process control charts to evaluate improvements in care processes.

**Results:**

EHR data from 9,621 patients with overweight and obesity were collected from 2021–2023. Providers documented elevated BMI in the problem list for at least 44% of eligible encounters and performed comorbidity screening in at least 15% of eligible encounters. Providers were more likely to perform problem list BMI documentation for older children, non-Hispanic Black children, children with public insurance, and children with a higher BMI percentile. Providers were also more likely to perform comorbidity screening among the same groups. The mean percentage of patients with an elevated BMI diagnosis on their problem list increased from 9.2% to 64% and the mean percentage of patients who had comorbidity screening labs performed increased from 7.8% to 32%.

**Conclusion:**

The implementation of the CDSS intervention was associated with an increased likelihood of delivering evidence-based care processes.

## 1. Introduction

Recent studies have estimated that about 1 in 6 children are overweight and almost 1 in 5 children have obesity [1]. Pediatric obesity has been linked to increased risk as an adult for obesity, type 2 diabetes (T2D), coronary heart disease, metabolic dysfunction-associated steatotic liver disease (MASLD), and cancer (e.g., leukemia, colorectal cancer, and breast cancer) [2–4]. These comorbidities highlight the importance of pediatric obesity as a global public health challenge [2, 5–7]. The American Academy of Pediatrics (AAP) and the U.S. Preventative Services Task Force (USPSTF) emphasize the necessity of BMI documentation; however, evidence shows that improved weight outcomes are driven by targeted interventions rather than documentation alone [8–10]. Since pediatric patients require regular monitoring of growth and development, clinicians have an opportunity to identify and address obesity during yearly well-child visits [11–13]. Healthcare providers face challenges in clinically managing pediatric obesity [7, 14, 15] thus could benefit from tools and support to provide evidence-based care [16–18].

Health information technology (HIT) has been leveraged to improve the quality of care in a variety of different disease areas, including diabetes, HIV, cardiovascular disease, kidney disease, and pulmonary disease [19, 20]. A clinical decision support system (CDSS) is defined as support that provides clinicians with knowledge and person-specific information in the electronic health record (EHR) workflow when needed. It can consist of several different tools, including clinical guidelines, OurPractice Advisories (OPAs) and reminders for providers, order sets, data reports, and documentation templates.

Although prior studies have used HIT tools to impact obesity care, many have been focused on adult populations [21] rather than pediatric. In previous pediatric settings, an OPA for pediatric obesity screening increased obesity diagnosis coding [22]. Similarly, another CDSS provider intervention with and without individualized family coaching found improvements in childhood body mass index (BMI) and obesity diagnosis coding [23, 24]. However, these interventions did not encourage problem list documentation nor comorbidity screening. The novelty of the study lies in not only initiating the documentation of elevated BMI and ordering comorbidity screening but also recommending intensive health behavior and lifestyle treatment (IHBLT), therefore aligning with recommended practices [8, 9].

The objective of our study was to conduct an observational real-world study of the effectiveness of HIT tools to support delivery of evidence-based care [8], such as problem list documentation and comorbidity screening, for childhood overweight/obesity.

## 2. Methods

### 2.1 CDSS Development

A multidisciplinary team of pediatricians (generalists and subspecialists) and nutrition researchers at an academic medical center reviewed the literature and current primary care practices for overweight/obesity management and developed revised guidelines focused on identifying children with elevated BMI and implementing screening follow-up for comorbidities, including T2D, MASLD, and hyperlipidemia. The group converged on the creation of a CDSS intervention which included an OPA and order set to support future population health management activities focused on overweight and obesity management. The OPA triggered for pediatric patients aged 2–17 years seen in primary care at a well-child or sick visit with a BMI percentile ≥ 85th who did not have an overweight or obesity related diagnosis or active pregnancy on their problem list. Once triggered, providers were alerted to document an elevated BMI on the patient’s problem list (“problem list BMI documentation”). The group elected to use BMI-based terminology for the problem list documentation (“BMI, pediatric, 85% to less than 95% for age”; “BMI, pediatric, 95–99% for age”; or “BMI, pediatric, > 99% for age”) following Centers for Disease Control and Prevention (CDC) BMI-for-age Growth Charts [12] rather than diagnoses with the words “overweight” or “obese” to avoid stigma. For patients aged 10–17 years who met the criteria, the OPA also triggered to remind providers to perform comorbidity laboratory screening. Providers were shown the most recent laboratory test date and result values for comorbidity screening if previously performed, and if labs were overdue, an order set was displayed that included the relevant screening labs for T2D (random glucose, hemoglobin A1c), MASLD (ALT, AST), and/or hyperlipidemia (lipid panel). Guidance on interpretation of test results and recommended follow-up actions based on abnormal labs were also developed (see supplementary materials). Finally, the order set allowed providers to document the delivery of nutrition and/or physical activity counseling and the inclusion of a healthy weight educational care guide in the after-visit summary.

### 2.2 CDSS Implementation

Prior to implementation of the OPA, written communication was circulated to leadership at 18 pediatric primary care clinics summarizing the purpose of the OPA, a detailed overview of how the OPA functions including the triggering criteria and prompts, and a summary of the comorbidity screening guidelines and recommended follow-up actions.

### 2.3 Evaluation of the Childhood Overweight/Obesity OPA

#### 2.3.1 Study Design, Setting, and Eligibility

We conducted a retrospective observational cohort study of the OPA implementation from August 25, 2021, to March 3, 2023, using two different approaches. First, we evaluated the population of children for whom the OPA fired. Since some children may have had multiple well-child visits during the observation period, we evaluated patient and provider characteristics of the cohort the first time that the OPA fired for a given patient. Second, we evaluated processes of care for the entire population of children in the clinic to examine the improvements in care over the study period.

Among the group for whom the OPA fired, separate outcomes of interest included provider action for problem list BMI documentation for the 2–17 year age group and provider action for comorbidity screening in the 10–17 year age group, which was defined as the ordering and completion of comorbidity laboratory tests. Covariates included sex, age category (2–5 years, 6–9 years, 10–13 years, and 14–17 years), race/ethnicity (non-Hispanic White, non-Hispanic Black, Hispanic, non-Hispanic Other, non-Hispanic Asian, and non-Hispanic Multiracial), primary insurance status (private and public), and BMI percentile category (85th −94th, 95th −98th, and ≥ 99th ). Logistic regression was conducted to examine the odds of problem list BMI documentation and comorbidity screening, adjusting for the covariates. A p-value < 0.05 was considered statistically significant for all statistical analyses.

We also created statistical process control charts (SPCCs) of the key measures of interest for all children who had a BMI percentile available based on weight and height measurement seen in pediatric primary care during the period September 2020 to March 2023. SPCCs [25] were created for the overall population and stratified for non-Hispanic White and non-Hispanic Black children. For the BMI documentation SPCC, the denominator consisted of the monthly number of children seen in pediatric primary care aged 2–17 years with a BMI percentile ≥ 85th and the numerator consisted of the monthly number of children with an elevated BMI on their problem list. For the comorbidity screening SPCC, the denominator consisted of the number of children seen in pediatric primary care aged 10–17 years with a BMI percentile ≥ 85th and the numerator consisted of the monthly number of children with comorbidity screening completed within the specified time frame of screening (i.e., two years prior or 30 days post the day of the visit). All analyses were conducted using R, version 4.1.2. The project was granted non-regulated human subjects research status by the local Institutional Review Board.

## 3. Results

### 3.1 Baseline Characteristics of Patients and Providers

Over the period, the OPA fired at an encounter for 10,835 children. We removed 1,214 patients who had missing or implausible values for BMI due to errors in data input, leaving a cohort of 9,621 patients.

Table 1 shows the demographics and clinical characteristics for the overall population of children with encounters for whom the OPA fired. In the overall study population, there were slightly more males (53.5%) than females and the most prevalent age group was 2–5 years (34.7%). The majority of participants were non-Hispanic White (61.5%), followed by non-Hispanic Black (14.9%). Almost two thirds of participants had private insurance (64.0%), and the majority (66.3%) of patients were overweight (BMI 85^th^-94^th^ percentile). Children were seen the most by providers in the General Pediatrics department (80.7%), most of whom were physicians (96.7%), and the OPA fired mostly during well-child visits (85.1%). Providers documented elevated BMI in the problem list for at least 44.9% of the eligible patient encounters in the overall population and performed comorbidity screening in at least 15.5% of the eligible patient encounters.

### 3.2 Main Outcomes Related to Overweight/Obesity OPA Use

Table 2 shows the outcomes of patients for whom the OPA fired. Providers were more likely to perform problem list BMI documentation for older children [Reference group 2–5 years; 6–9 years OR 2.33 (95% CI: 2.08, 2.61); 10–13 years OR 2.70 (95% CI: 2.41, 3.03); and 14–17 years OR 2.28 (95% CI: 1.97, 2.66)]; for non-Hispanic Black children [OR 1.24 (95% CI: 1.09, 1.42)] compared with non-Hispanic White children, with no significant differences for other race/ethnicity groups; and for children with public insurance [OR 1.49 (95% CI: 1.35, 1.64)] compared with children with private insurance. Providers were also more likely to perform problem list BMI documentation for children with a higher BMI percentile [Reference group ≥85^th^-94^th^; 95^th^-98^th^ OR 1.71 (95% CI: 1.55, 1.89); ≥99^th^% OR 4.80 (95% CI: 3.91, 5.92)]. Family medicine physicians [OR 0.57 (95% CI: 0.49, 0.67)] and nurse practitioners [OR 0.63 (95% CI: 0.41, 0.97)] were less likely to perform problem list BMI documentation compared with general pediatric physicians; and all providers were much less likely to take action at other visits [OR of 0.14 (95% CI: 0.12, 0.16)], compared with well-child visits. There were no statistically significant differences in BMI documentation by sex.

Providers were more likely to perform comorbidity screening for older children 14–17 years [OR 1.41 (95% CI: 1.16, 1.72)] compared with children 10–13 years, for non-Hispanic Black children [OR 1.41 (95% CI: 1.10, 1.80)] compared with non-Hispanic White children, and for children with public insurance [OR 1.67 (95% CI: 1.38, 2.03)] compared with children with private insurance.

Providers were more likely to perform comorbidity screening for children with a higher BMI [Reference group ≥85^th^-94^th^; 95^th^-98^th^ OR 1.68 (95% CI: 1.37, 2.06); ≥99^th^ OR 3.76 (95% CI: 2.74, 5.15)]. There were no statistically significant differences in comorbidity screening by sex, department, or provider type. Additionally, providers were less likely to perform comorbidity screening at other visits [OR 0.61 (95% CI: 0.46, 0.80)] compared with well-child visits.

Regardingthe types of labs completed, a total of 562 patients had laboratory testing done, with many undergoing multiple tests. Most participants underwent lipid panel testing, with 87.2% (n=490) having their cholesterol levels assessed. ALT (alanine aminotransferase) and AST (aspartate aminotransferase) testing were conducted in 75.8% (n=426) and 75.1% (n=422) of individuals, respectively. Hemoglobin A1c and glucose testing were measured in 65.3% (n=367) and 52.1% (n=293) of individuals, respectively.

**Figure 1:**
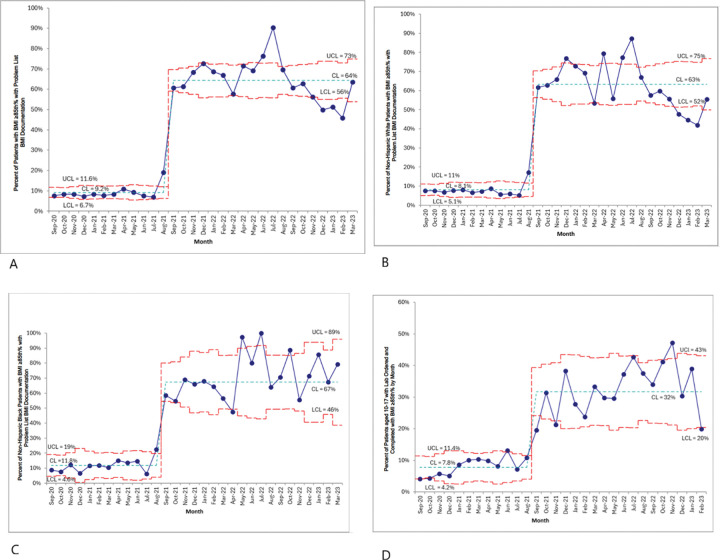
**a** SPC chart showing the percentage of patients with BMI ≥85^th^ percentile seen monthly between September 2020 and March 2023, who had problem list BMI documentation. Abbreviations: BMI: body mass index; CL: control limit; LCL: lower control limit; SPCC: statistical process control chart; UCL: upper control limit. **b** SPC chart showing the percentage of Non-Hispanic White patients with BMI ≥85^th^ percentile seen monthly between September 2020 and March 2023, who had problem list BMI documentation. Abbreviations: BMI: body mass index; CL: control limit; LCL: lower control limit; SPCC: statistical process control chart; UCL: upper control limit. **c** SPC chart showing the percentage of Non-Hispanic Black patients with BMI ≥85^th^ percentile seen monthly between September 2020 and March 2023, who had problem list BMI documentation. Abbreviations: BMI: body mass index; CL: control limit; LCL: lower control limit; SPCC: statistical process control chart; UCL: upper control limit. **d** SPC chart showing the percentage of patients with BMI ≥85^th^ percentile seen monthly between September 2020 and March 2023, who completed comorbidity screening. Abbreviations: BMI: body mass index; CL: control limit; LCL: lower control limit; SPCC: statistical process control chart; UCL: upper control limit.

The SPCC in Figure 1a shows that in the observation period before the OPA was in action, the mean percentage of patients with an overweight/obese diagnosis on their problem list was 9.2%. Following the implementation of the OPA in August 2021, the mean percentage increased to 64% by the end of the period. Figure 1b and Figure 1c display BMI documentation rates in non-Hispanic White and non-Hispanic Black patients, respectively. Once the OPA was in action, documentation rates remained comparable between racial groups in the months following implementation, with mean rates of 67% for non-Hispanic Black patients and mean rates of 63% for non-Hispanic White patients. The SPCC in Figure 1d shows that in the observation period before the OPA was in action, the mean percentage of patients who had comorbidity screening labs performed was 7.8%. After the time of implementation of the OPA in August 2021, the mean percentage increased to 32% by the end of the period. A similar increase in comorbidity screening lab completion was seen for both non-Hispanic White and non-Hispanic Black children.

## 4. Discussion

This study demonstrated that successful implementation of a CDSS improves adherence to evidence-based guidelines for overweight and obese children. We found an overall increase in problem list BMI documentation from 9.2% to 64% among overweight and obese pediatric patients, and an increase in comorbidity screening from 7.8% to 32%, a significant improvement in evidence-based processes of care for the health system. Strengths of the study included the alignment of the CDSS to health system guidelines, implementation across a large-scale health system of 18 pediatric clinics and 275 providers, and a representatively diverse sample size of patients. The CDSS is still currently used in the health system with outcomes continuously measured to ensure adherence to the guidelines [26] and support further improvement.

We found a higher rate of providers taking action for non-Hispanic Black children, and children covered by public insurance, even after accounting for overweight/obesity rates. This indicates a more reliable quality of care being delivered, which is crucial given the disproportionate burden of overweight and obesity in these populations [27]. Despite the OPA triggering universally for all weight and age groups, the fact that providers were more likely to take action for more severely obese children, and less likely to take action for younger children, especially the 2–5 years age category, indicates the influence of provider judgment on adherence to guidelines. We speculate that providers are potentially more worried about children with a greater severity of obesity leading to more documentation. Meanwhile, providers may be reluctant to perform problem list BMI documentation for younger children due to a potential fear of labeling or weight stigmatization at an early age.

Our findings regarding BMI diagnoses are consistent with the results of previous studies. Ayash et al. found that after implementing an OPA and order set, physicians increased documentation of an ICD-9 obesity diagnosis from 17.3% to 38.0%, however this OPA only targeted obese pediatric patients, was implemented back in 2006–2008, and was only associated with visit diagnoses not the problem list [22]. Similar to our study findings, they found that non-Hispanic Black children were more likely to have an obesity diagnosis compared with non-Hispanic White children, and younger children were less likely to have an obesity diagnosis compared with children 12–18 years. Taveras et al. [24] also implemented an OPA for elevated BMI documentation in the visit, reporting an increase in documentation for the visit diagnoses from ~58% to ~74%. However, their study focused solely on obese and not overweight children, capturing a smaller population as the study was conducted during a clinical weight management trial back in 2013–2014. Shaikh et al. [28] also documented an increase in elevated BMI documentation with an OPA from 40% to 57% for overweight and obese children but obtained outcomes through medical record reviews of a smaller sample size of approximately 450 patients between 2008 and 2010.

The literature on the impact of CDSS on comorbidity screening is mixed. Consistent with our study, Shaikh et al. [28] found that lab orders for assessment of dyslipidemia and diabetes increased from 17% to 27% (p <0.001) for overweight and obese children, but again outcomes were captured through medical record reviews with a small sample size from an older time period. Our results for comorbidity screening contrast with a study by Kaufman et al. [29], which implemented a CDSS tool to recommend screening for dyslipidemia, MASLD, and T2D for children aged 7–18 years who were overweight with a family history of diabetes or were obese. Through chart review comparing screening performance in 2009 with performance after implementation in 2011 and 2013, they found no increase in the rates of screening for these obesity-related comorbidities. Their CDSS tool did not include an order set with links to recommended tests, which may have impeded adoption, and in addition, their study population had a low proportion of patients from racial and ethnic minority groups.

Our study extends the existing literature by targeting problem list BMI documentation rather than solely focusing on encounter-level diagnosis codes. This distinction is important because the problem list offers a unique opportunity for population management to improve patient care. Unlike encounter diagnoses, which are only tied to specific orders or visits, the problem list provides a concise and consistent summary of a patient’s ongoing chronic illnesses and is available to all providers caring for the patient across all healthcare interactions, allowing for more effective tracking and management of disease. The problem list allows for greater opportunities for population management, similar to other chronic diseases such as diabetes, and may facilitate better coordination among health care providers to manage pediatric obesity more effectively. As a result of having the diagnosis on the problem list, our health system has implemented additional OPAs that prompt providers to offer other types of weight-related interventions for children with elevated BMI.

Limitations of our study include errors in measurement of the height or weight of the individual. First, if labs were performed externally and are not in the health system, they may falsely trigger an OPA, but most children seen in primary care would likely use the internal laboratory system. Second, for comorbidity screening we opted to define the measure as labs ordered and completed, but we acknowledge that physicians may have ordered labs for their patients without completion of the labs, leading to an underestimation of adherence to guidelines. Third, we recognize that the timeframe of the study period included the COVID-19 pandemic, which may have impacted clinical volumes and a reprioritization of health issues on behalf of families. Fourth, BMI may misclassify individuals as overweight and obese as it is not a gold-standard for body fat measurement, and this may lead to provider reluctance in assigning a diagnosis or ordering labs.

Our finding that the family physicians and family and pediatric nurse practitioners were less likely to take action with the problem list BMI documentation warrants further study and exploration. One possible explanation is that the department did not prioritize the documentation of pediatric obesity and overweight as a quality metric at the time, resulting in less targeted education on this topic. However, it is important to note that there was no difference in comorbidity screening between these groups. Furthermore, given that few providers took action outside of a well-child visit, it will be important to remove the OPA triggering outside of the well-child visit to reduce unnecessary notifications to providers.

## 5. Conclusion

The CDSS intervention was associated with an increased likelihood of delivering evidence-based care processes. The CDSS developed for this intervention is a component of a larger initiative focused on using a learning health system (LHS) framework to positively affect obesity outcomes. We included the SPCC analyses to demonstrate that the CDSS resulted in a clear and sustained improvement in evidence-based processes of care for children who are overweight and obese. Health information technology tools are vital for ensuring the reliability and persistence of evidence-based care processes.

## Supplementary Material

Supplementary Files

This is a list of supplementary files associated with this preprint. Click to download.

• SupplementalMaterials.docx

## Figures and Tables

**Table 1:** Participant characteristics for whom the OPA for problem list BMI documentation and comorbidity screening was shown to the provider.*

	Ages 2–17 years			Ages 10–17 years		
		*Action*			*Action*	
	Overall N (%)	BMI Documentation N (%)	No BMI Documentation N (%)	Overall N (%)	Labs Completed N (%)	No Labs Completed N (%)
**Number of Participants**	9,621 (100%)	4,319 (44.9%)	5,302 (55.1%)	3,618 (100%)	562 (15.5%)	3,056 (84.5%)
***Sex***											
Male	5,150 (53.5%)	2,349 (45.6%)	2,801 (54.4%)	1,892 (52.3%)	289 (15.3%)	1,603 (84.7%)
Female	4,471 (46.5%)	1,970 (44.1%)	2,501 (55.9%)	1,726 (47.7%)	273 (15.8%)	1,453 (84.2%)
***Age Category***											
2–5 years	3,341 (34.7%)	1,109 (33.2%)	2,232 (66.8%)	NA	NA	NA
6–9 years	2,662 (27.7%)	1,364 (51.2%)	1,298 (48.8%)	NA	NA	NA
10–13 years	2,494 (25.9%)	1,334 (53.5%)	1,160 (46.5%)	2,494 (68.9%)	361 (14.5%)	2,133 (85.5%)
14–17 years	1,124 (11.7%)	512 (45.6%)	612 (54.4%)	1,124 (31.1%)	201 (17.9%)	923 (82.1%)
***Primary Insurance***											
Private	6,155 (64.0%)	2,543 (41.3%)	3,612 (58.7%)	2,308 (63.8%)	286 (12.4%)	2,022 (87.6%)
Public	3,466 (36.0%)	1,776 (51.2%)	1,690 (48.8%)	1,310 (36.2%)	276 (21.1%)	1,034 (78.9%)
***Race/Ethnicity***											
Non-Hispanic White	5,915 (61.5%)	2,524 (42.7%)	3,391 (57.3%)	2,258 (62.4%)	296 (13.1%)	1,962 (86.9%)
Non-Hispanic Black	1,433 (14.9%)	764 (53.3%)	669 (46.7%)	576 (15.9%)	126 (21.9%)	450 (78.1%)
Hispanic	764 (7.9%)	346 (45.3%)	418 (54.7%)	300 (8.3%)	50 (16.7%)	250 (83.3%)
Non-Hispanic Asian	520 (5.4%)	231 (44.4%)	289 (55.6%)	193 (5.3%)	30 (15.5%)	163 (84.5%)
Non-Hispanic Other	496 (5.2%)	239 (48.2%)	257 (51.8%)	148 (4.1%)	30 (20.3%)	118 (79.7%)
Non-Hispanic Multiracial	493 (5.1%)	215 (43.6%)	278 (56.4%)	143 (4.0%)	30 (21.0%)	113 (79.0%)
***BMI Percentile Category***											
85th-94th	6,376 (66.3%)	2,491 (39.1%)	3,885 (60.9%)	2,408 (66.6%)	292 (12.1%)	2,116 (87.9%)
95th-98th	2,639 (27.4%)	1,383 (52.4%)	1,256 (47.6%)	998 (27.6%)	192 (19.2%)	806 (80.8%)
≥ 99th	606 (6.3%)	445 (73.4%)	161 (26.6%)	212 (5.9%)	78 (36.8%)	134 (63.2%)
***Department***											
General Pediatrics	7,760 (80.7%)	3,593 (46.3%)	4,167 (53.7%)	2,796 (77.3%)	451 (16.1%)	2,345 (83.9%)
Family Medicine	1,310 (13.6%)	439 (33.5%)	871 (66.5%)	597 (16.5%)	73 (12.2%)	524 (87.8%)
Internal Medicine - Pediatrics	551 (5.7%)	287 (52.1%)	264 (47.9%)	225 (6.2%)	38 (16.9%)	187 (83.1%)
***Provider Type***											
Physician	9,302 (96.7%)	4,227 (45.4%)	5,075 (54.6%)	3,473 (96.0%)	549 (15.8%)	2,924 (84.2%)
Nurse Practitioner	141 (1.5%)	35 (24.8%)	106 (75.2%)	62 (1.7%)	7 (11.3%)	55 (88.7%)
Physician Assistant	178 (1.9%)	57 (32.0%)	121 (68.0%)	83 (2.3%)	7 (11.3%)	55 (88.7%)
***Appointment Type***											
Well Child Exam	8,190 (85.1%)	4,116 (50.3%)	4,074 (49.7%)	2,981 (82.4%)	492 (16.5%)	2,489 (83.5%)
Not a Well Child Exam	1,431 (14.9%)	203 (14.2%)	1,228 (85.8%)	637 (17.6%)	70 (11.0%)	567 (89.0%)

Abbreviations: BMI: body mass index; OPA: OurPractice Advisory.

* The overall columns represent column percentages and the action columns represent the proportion of action taken for each group.

**Table 2:** Odds of taking action for whom the OPA for problem list BMI documentation and comorbidity screening was shown to the provider.

	Odds Ratio for problem list BMI documentation OR (95% CI)	P-Value	Odds Ratio for Comorbidity Screening OR (95% CI)	P-Value
**Number of Participants**	9,621		3,618	
***Sex***				
Female	Ref		Ref	
Male	1.06 (0.97, 1.15)	0.2	0.88 (0.73, 1.07)	0.2
***Age Category***				
2–5 years	Ref		NA	
6–9 years	2.33 (2.08, 2.61)	<0.001	NA	
10–13 years	2.70 (2.41, 3.03)	<0.001	Ref	
14–17 years	2.28 (1.97, 2.66)	<0.001	1.41 (1.16, 1.72)	<0.001
***Primary Insurance***				
Private	Ref		Ref	
Public	1.49 (1.35, 1.64)	<0.001	1.67 (1.38, 2.03)	<0.001
***Race/Ethnicity***				
Non-Hispanic White	Ref		Ref	
Non-Hispanic Black	1.24 (1.09, 1.42)	0.001	1.41 (1.10, 1.80)	0.007
Hispanic	1.03 (0.87, 1.22)	0.7	1.17 (0.82, 1.63)	0.4
Non-Hispanic Asian	1.15 (0.94, 1.39)	0.2	1.39 (0.90, 2.08)	0.12
Non-Hispanic Other	1.05 (0.86, 1.28)	0.6	1.46 (0.93, 2.22)	0.088
Non-Hispanic Multiracial	0.93 (0.76, 1.14)	0.5	1.50 (0.95, 2.28)	0.071
***BMI Percentile Category***				
85th-94th	Ref		Ref	
95th-98th	1.71 (1.55, 1.89)	<0.001	1.68 (1.37, 2.06)	<0.001
≥ 99th	4.80 (3.91, 5.92)	<0.001	3.76 (2.74, 5.15)	<0.001
***Department***				
General Pediatrics	Ref		Ref	
Family Medicine	0.57 (0.49, 0.67)	<0.001	0.75 (0.56, 1.01)	0.063
Internal Medicine - Pediatrics	1.07 (0.89, 1.29)	0.5	1.03 (0.70, 1.49)	0.9
***Provider Type***				
Physician	Ref		Ref	
Nurse Practitioner	0.63 (0.41, 0.97)	0.038	0.92 (0.36, 2.00)	0.8
Physician Assistant	0.91 (0.62, 1.32)	0.6	0.47 (0.18, 1.06)	0.10
***Appointment Type***				
Well Child Exam	Ref		Ref	
Not a Well Child Exam	0.14 (0.12, 0.16)	<0.001	0.61 (0.46, 0.80)	<0.001

Abbreviations: BMI: body mass index; OPA: OurPractice Advisory.

## Data Availability

The datasets used and/or analyzed during the current study are available from the corresponding author on reasonable request.
